# Factors of balance determining the risk of falls in physically active women aged over 50 years

**DOI:** 10.7717/peerj.12952

**Published:** 2022-02-15

**Authors:** Grzegorz Bednarczuk, Izabela Rutkowska

**Affiliations:** Department of Movement Teaching, Faculty of Rehabilitation, Józef Piłsudski University of Physical Education, Warsaw, Poland

**Keywords:** Balance, Risk of falls, Elderly

## Abstract

**Background:**

Balance disorders are believed to be one of the main reasons for falls in older adults. They are related to natural processes of ageing, resulting in deterioration of information integration and processing from the vestibular, somatosensory and visual systems. The consequence is an increased number of postural sways, which are some of balance factors. Balance control in static and dynamic activities is an essential element of daily functioning of older citizens. It seems that balance assessment is essential to determine the risk of falls, as well as to determine which factors of balance have greatest impact on the risk of falls.

**Methods:**

The study involved physically active female students (*n* = 36, mean age 67,11 ± 5,35) of a University of the Third Age. We used the Balance System SD platform to assess their balance in four tests with eyes open and with eyes closed and to determine the risk of falls. We assessed the relationships between individual balance indices (overall stability index, anterior/posterior stability index, medial/lateral stability index) and the falls risk index. We also determined those factors which predicted the risk of falls the most.

**Results:**

The studied subjects had low risk of falls for their age category. In most measurements there were relationships between the risk of falls and the size of sways in the coronal plane and the overall stability index. We also found that the overall stability index calculated in measurements with eyes closed predicted the risk of falls of the studied physically active females most accurately (R^2^ 0.391 F(1.34)=23.475; <0.000). The subjects were physically active and their falls risk index was low - this allowed us to presume that there was a relationship between these two factors. Preventive programmes should include exercise performed with eyes closed, and tests conducted with eyes closed seem to be most sensitive in determining balance disorders in physically active women.

## Introduction

The process of ageing is accompanied by incremental degeneration of all the functional and autonomous systems. The degeneration is the effect of the local and general ageing of the tissues and of the slowdown of the biological, metabolic and regeneration processes. With age we can observe gradual impairment of the function of the motor and postural systems; the systems on whose function the postural stability depends (Błaszczyk & Czerwosz, 2005). The main symptoms of postural instability are balance disorders resulting in falls. Balance disorders develop approximately at the age of 50–60 years, intensify with age (Błaszczyk & Czerwosz, 2005) and may affect quality of life (Leś et al., 2019). In subjects in their eighties the issue of falls affects more than 30% of population, and most falls happen after tripping (Błaszczyk & Czerwosz, 2005).

Intraindividual factors related to the risk of falls in older adults include visual, balance and muscle strength disorders. Intraindividual factors may be divided into modifiable factors (illness, poor balance, gait disorders, visual deficits) and non-modifiable factors (age, female sex, race, chronic diseases of the locomotor system, chronic mental illnesses). Falls are more common in women than in men. This is related to women’s lower physical activity, poorer muscle strength (Fabre et al., 2010), as well as to higher requirements towards them in respect of *e.g.*, activities of daily living (Boelens, Hekman & Verkerke, 2016). The somatosensory, vestibular and visual systems are responsible for providing information for the nervous system and for maintaining balance. The processes of ageing lead to dysfunctions of these systems, as well as affect the quality of integration and processing of information sent by these systems. Consequently, they lead to an increased number of postural sways. Postural sways are some of the static balance factors (Fabre et al., 2010). Balance control in static and dynamic activities is an essential element of daily functioning of older adults. The degenerative changes which develop in the process of ageing affect negatively the perception of the environment and of the body. The decreased velocity of information transfer in the central nervous system and the delayed reaction of the muscle system may result in losing balance in numerous situations. The falls resulting from loss of balance constitute a serious issue and are related to increased mortality and to the necessity of hospitalization of older adults. The fear of falls is identified as an independent factor resulting in disability, lowered quality of life and decreased independence and functioning of older adults (Sofianidis, Dimitriou & Hatzitaki, 2017).

The level of functional abilities of older adults is affected by the functioning of their cardiorespiratory, osteorarticular and nervous systems. Components that are related with their function are motor abilities such as strength, endurance, balance, coordination, limberness and body composition (Fabre et al., 2010). Risk of falls factors related to poor functional abilities observed in older adults include i.a. poor balance levels, limberness, reactive power, muscular strength and physical activity, problems with locomotion, problems with transfers, gait disorders (feet lifted too low, feet placed incorrectly, too slow gait), and also haste in performing various tasks (Allison, Brooke-Wavell & Folland, 2018; Boelens, Hekman & Verkerke, 2016; Kobayashi et al., 2006; Moreira et al., 2017; Olson, Chen & Wang, 2011; Patil et al., 2015; Sofianidis et al., 2009). The above mentioned mobility disorders (including gait, rotations, changing body position) result from increasing unsteadiness, slow movements, and also from decreased joint mobility in lower extremities and deformation to the feet and accompanying pain (Bergland, 2012; Patil et al., 2015; Twardowska-Rajewska, 2006). Old age-related pathophysiological changes predisposing for falls include first of all the deterioration of the functioning of the nervous, motor and visual systems, *i.e.,* the systems responsible for the coordination, gait and balance (Ołdak et al., 2013).

Human postural control is also influenced by genetic factors—primitive reactive strategies. One of such strategies is the strategy of the ankle joint, which helps to maintain balance by making movements in the ankle joint. Another strategy is the strategy of the hip joint, which—similarly—is responsible for the movements in the hip joint. There is also the step strategy—in this strategy, making a step restores balance. In case of older adults balance disorders may be observed in gait or in getting up from seated position (Winiarska et al., 2017). Maintaining balance involves therefore using a number of strategies aimed at maintaining the centre of gravity in the base of support when standing or when moving (static and dynamic balance) (Olson, Chen & Wang, 2011). However, older adults experience falls when standing, changing position, or during locomotion, in situations in which the influence of environmental factors exceeds a person’s current physical and functional abilities (Moreira et al., 2017).

The literature review shows that balance disorders are believed to be one of the main reasons for falls in older adults. Significant risk factors for falls include: deficits in systems that safeguard maintaining balance and postural stability, slowdown of reaction time, and deterioration of the function of the lower extremities (Ołdak et al., 2013). Maintaining balance requires integrating a large amount of information generated by several systems (the vestibular, somatosensory, visual or muscular systems). A measurement of balance is therefore the quality of function of the whole system responsible for maintaining balance (Muir et al., 2010). Balance and gait indices can be used to predict falls. The risk of falls increases with the difficulty of the task which requires stabilization or balance, particularly in the coronal plane. The length, frequency and velocity of gait impact the risk of falls, too (Bergland, 2012). The falls risk index may be used as a measure of stability (the lower its value the better body stability). A correlation therefore should be expected between this index and balance levels (Terlecka & Ostrowska, 2014). In double leg stance, subjects without dysfunctions make slight movements the centre of foot pressure on the ground (CoP) in antero-posterior and medio-lateral dimensions. Healthy subjects are able to maintain the CoP within the limits of the base of support, therefore avoiding falls (Eysel-Gosepath et al., 2016). A greater involvement of the system of balance control can be observed in dynamic tests, which seem to be more relevant and to allow for earlier diagnosis of disorders in comparison to static tests (Wiszomirska et al., 2013).

The literature suggests a relationship between the risk of falls and balance level, as the higher the risk the poorer the balance. It seems that an appropriate level of balance can help reduce the number of falls in the elderly. Adequate control and body stabilization in *e.g.*, locomotion or in activities of daily living can solve the problem of balance disorders in patients over 50 years of age. Is it possible therefore to indicate which of the factors determining balance levels, even at low risk of falls, decides about it to the greatest extent? An answer to this question may be provided by a detailed assessment of balance, involving its changeable conditions, such as unstable surface or excluding one of the receptors responsible for maintaining balance.

The aim of the study therefore was to determine the factors of balance which predict the risk of falls with highest accuracy in physically active women aged over 50 years. Such information may extend the scope of action related to falls prevention, as well as provide for efficient planning and designing therapeutic activities related to risk of falls or consequences of falls.

## Materials & Methods

### Study participants

The study involved female students (*n* = 36) of the University of the Third Age of the University of Physical Education in Warsaw with a minimum of two year participation (Table 1). The curriculum of the University includes classes which rely on physical activity. This allows for an assumption that the subjects are physically active. The subjects were informed about the procedures of the study, and about the fact that they could withdraw from the study at any of its stages. The study obtained an approval from the Senate Ethics Committee for Scientific Research of the University of Physical Education in Warsaw (SKE 01-60/2017). All participants gave written informed consent after a detailed written and oral explanation of the risk and benefits resulting from participation in this study, as outlined in the Declaration of Helsinki (2008). On the basis of this information, the subjects expressed informed consent to participate in the study.

**Table 1:** Characteristics of the study population (*n* = 36).

	Age (years)	Body mass [kg]	Height [cm]
	67.11 ± 5.35	64.57 ± 7.01	160.08 ± 6.32
Median	67	63.0	160
Min–Max	54–80	54.3–78.0	150–178

### Study procedure

The measurements were carried out four times during a single semester at the University of the Third Age (at the beginning of March, at the end of April, in the middle of May, and at the end of June). To exclude injuries to the balance system, the subjects underwent static and dynamic tests: the Unterberger, Babiński Weill and Romberg tests (Kulma, 2009). The subjects tested negatively in all the trials. The subjects did not declare suffering from any orthopaedic or pain disorders which would make it impossible for them to participate in the measurements. For the measurements, we used the Balance System SD platform from Biodex, connected to the Medical System Biodex Inc. software. The software allows for conducting measurements of the inclination angle in every axis. For the analysis, we used the Overall Stability Index (OSI), the Anterior/Posterior Stability Index (APSI), Medial/Lateral Stability Index (MLSI), and the Fall Risk Index (FRI). A high value of an index denotes high sways of the body and therefore poorer balance and greater risk of falls. The following tests were taken on the Biodex platform:

1. Double leg stance with eyes open on stable platform,
2. Double leg stance with eyes closed on stable platform,
3. Falls Risk Test on unstable platform.

Each of the tests took 3 × 20 s. The pause between every single repetition was 10 s. During the Falls Risk Test, the stability of the platform was reduced from level 6 to level 2. The full scales has 12 levels (Balance System SD, 0000).

### Methods of statistical analysis

To conduct the statistical calculations, we used the STATISTICA programme version 13 (TIBCO Software Inc.; Palo Alto, CA, USA). We used the Shapiro–Wilk test to determine normal distribution. As the distribution did not meet the normal criteria, the data was logarithmized (normal logarithm) (Curran-Everett, 2018). To establish the relationships between the Risk of Falls (FRI) and the balance indices: Overall Stability Index (OSI), Anterior/Posterior Stability Index (APSI) and Medial/Lateral Stability Index (MLSI), we calculated the Spearman’s rho rank correlation coefficient, interpreted according to the values: 0.00–0.19 very weak, 0.20–0.39 weak, 0.40–0.59 moderate, 0.60–0.79 strong and 0.80–1.0 very strong. We conducted the analysis of backward stepwise regression to determine which index had the greatest impact on the risk of falls (FRI). As explanatory (descriptive) variables we used each time the balance indices OSI, APSI, MLSI, taken in tests with eyes open and eyes closed. The coefficient of determination (R^2^) was calculated for factor that most accurately predicted risk of falls. We set statistical significance at *p* < 0.05.

## Results

### Relationships between the analysed balance indices

The level of falls risk index in the first measurement was not statistically significantly correlated with any of the analysed balance indices. Positive weak correlation that suggested a tendency (*ρ* = 0.321; *p* = 0.056) was observed only for the overall stability index (OSI) with eyes closed. We did not find relationship between falls risk and stability indices with eyes open or the remaining stability indices with eyes closed.

In the second measurement we found a statistically significant positive weak correlation between falls risk and medial/lateral stability index (MLSI) with eyes open (*ρ* = 0.346; *p* =0.039). For the values of remaining correlation coefficients, tendency was not observed.

The correlation coefficient between the falls risk and the medial/lateral stability index (MLSI) with eyes open (*ρ* = 0.398; *p* = 0.016) had higher value in the third measurement. In addition, there was a positive weak relationship that suggested tendency (*ρ* = 0.317; *p* = 0.060) with the overall stability index (OSI) with eyes closed. Therefore, in the third measurement the falls risk index was greater with poorer medial/lateral stability control, especially with eyes open.

Two indices of stability control with eyes open showed significant and moderate relationship with falls risk in the fourth measurement (MLSI: *ρ* = 0.560; *p* <‘0.001; OSI: *ρ* = 0.518; *p* = 0.001), and for the relationship of the APSI with falls risk a tendency was observed (*ρ* = 0.310; *p* = 0.066).

In the measurement with eyes closed we also observed significant correlations of a similar strength of the analysed indices with falls risk (MLSI: *ρ* = 0.451; *p* = 0.006; OSI: *ρ* = 0.472; *p* = 0.004).

### Regression analysis

The result obtained in the first, the third and the fourth measurement allowed for predicting risk of falls in 22, 25 and 24%, respectively (Table 2) on the basis of the overall stability index (OSI) with eyes closed. The risk of falls is greater with greater values of this index. The coefficient of determination (R^2^) in the analysis which considers the values of all the measurements of overall stability index with eyes open rose to 0.391, which means that it explains the risk of falls in almost 40%.

**Table 2:** Predictors of falls risk in subsequent measurements for Overall Stability Index determined with eyes closed –backward regression analysis results.

Survey	Beta	*t*	*p*	R^2^	F	*p*
1st	0.494	3.318	<0.002	0.222	F (1 34) = 11.010	<0.002
3rd	0.521	3.564	<0.001	0.250	F (1 34) = 12.707	<0.001
4th	0.509	3.451	<0.001	0.237	F (1 34) = 11.913	<0.001
x	0.639	4.845	<0.001	0.391	F (1 34) = 23.475	<0.001

## Discussion

The aim of the study focused on determining the factors of balance which may most accurately predict the risk of falls in physically active women aged over 50 years. The method we used allowed us to determine the overall stability index (OSI), sways in the coronal plane (MLSI) and in the sagittal plane (APSI). The falls risk index is the resultant of the collected data. This means it should denote comprehensive information on balance. In gait there are movements of the lower extremity girdle i.a. medial and anterior/posterior. The main findings of of our survey indicate the importance of the movements control in the coronal plane in relation to the risk of falls, and the overall stability index determined with eyes closed to the greatest extent predicted the risk of falls.

As most falls happen during locomotion, it seems justified to search for relationships between them and control of sways in these planes. A questionnaire administered to 56 subjects (72% women) who had experienced falls within 6 months confirmed that falls happened mainly as a result of slipping (54% of responses), usually while walking (48% of responses) and walking upstairs and downstairs (18% of responses) (Gazibara et al., 2017). The subjects who experience falls often declare balance disorders during locomotion (Joyce et al., 2020).

In the first measurement only a tendency was observed between the falls risk and the overall stability index with eyes closed - this may point to the impact of sight on the process on maintaining balance. The next measurement revealed a positive weak relationship between the falls risk and balance control in the coronal plane with eyes open. This may point to the impact of balance control during movements performed in this plane. The third measurement seems to confirm the above conclusions, as the relationship power increased between the falls risk and the medial/lateral stability index. The measurement taken at the end of the semester (the fourth measurement) also pointed to a significant moderate relationship between the falls risk with the medial/lateral stability index both in tests with eyes open and with eyes closed. These tests showed a significant positive moderate relationship between the index of the risk of falls and the overall stability index. Our findings seem to confirm the conclusions drawn by Wojciechowska-Maszkowska (2007). Her subjects who had high risk of falls had high values of sways in the coronal plane. Wojciechowska-Maszkowska (2007) observed that postural stability decreased with age, particularly in the coronal plane; and she also noted that there were significant differences in postural stability among her individual oldest subjects. Her older subjects loaded their lower extremities asymmetrically when standing and the author believed that it allowed for the shortening of reaction time needed to react to distortions to balance. When older adults fall, most of them fall forward (60%) while moving, and a result of tripping (Błaszczyk & Czerwosz, 2005). Our study found good balance control of the subjects in the sagittal plane. This may be proved by the fact that there is no relationship of this index with the generally low falls risk index in the studied age group. The mean value of four measurements of falls risk index was 1.74 ± 0.29, with the norm at the level of 1,4–3,4 for subjects aged 54–89 years (Balance System SD, 0000). Vanderhoek, Coupland & Parkhouse (2000) explained dynamic balance improvement in their patients with improvement of power of knee flexors and extensors and hip flexors. This suggests that muscle control of movements in the sagittal plane may result in lowering the risk of falls in older adults. We have to stress that our study subjects were physically active women, aware of the impact of physical activity on their functional state. The analysis of their measurements, which focused on relationships of stability indices with the falls risk index, explains why falls risk was so low in the studied population. Thus, improving the level of balance parameters may have an impact on reducing the risk of falls.

The relationships of the analysed stability indexes with the falls risk observed in our study, may suggest their impact on the falls risk. Older adults (aged approximately 71 years) qualified to the low falls risk group had lower balance levels expressed with time of maintaining stable posture on the right and on the left leg and shorter functional reach excursion (cm) in comparison to subjects who did not experience falls. Subjects who had high falls risk had significantly poorer results of the above mentioned tests in comparison to subjects who did not experience falls (Moreira et al., 2017). In their study Batcir & Melzer (2018) assumed that there may be a relationship between high values of stability indices, such as sways to the front, to the back and to the sides, and falls risk. Their study confirmed this assumption, and it also pointed to the significantly better balance of physically active subjects (cyclists) in comparison to physically inactive subjects. The active subjects controlled body sways to every side better (particularly side sways), which may have an impact on a significantly lower risk of falls.

The presence of relationships between the analysed stability indices and falls risk allowed us to assume that these indices (OSI, APSI, MLSI) may be predictors of falls. It turned out that the factor that most accurately predicted risk of falls in our subjects was the overall stability index in tests with eyes closed. This was confirmed in the first, third and fourth measurements, and in the total data from all four measurements. An attempt at specifying falls risk predictors in our study found that all explanatory variables were positive predictors, and that therefore one may expect that high risk of falls accompanies high values of such variables. It turned out that regardless of the time at which the measurements were taken, the risk of falls in studied subjects can be anticipated on the basis of the overall stability index in test with eyes closed. For three of the measurements, and for the mean of all the four measurements this was a statistically significant predictor. The importance of accurate assessment of individual motor skills in determining the level of functional abilities of i.a. elderly, was emphasized by Forte, Neiva & Marinho (2021). Our results seems to be confirmed by opinions of physiotherapists—they believe that high risk of falls can be predicted when there are low overall stability and problems with locomotion (91% respondents) (Kalu, Vlachantoni & Norman, 2019). It turns out that the fear associated with awareness of poor balance also increases the risk of falls (Monteiro et al. 2019). In their study, Terlecka & Ostrowska (2014) found a relationship (*ρ* = −0.48), though not statistically significant (*p* < 0.07) between the subjective assessment of balance and the risk of falls in older females. Subjects who declare fewer falls or no falls at all have good balance levels. Results of the following tests: up and go, chair stand, and step in place predicted risk of falls in older adults most precisely (Tongterm et al., 2015). It is worth to stress that sagittal plane moves dominate in these tests. Our participants had low risk of falls and it was not predicted by the anterior/posterior stability index—this may point to good postural control in this plane.

Results of conducted analyses did not always point to significant relationships between falls risk index and individual indices of balance levels (OSI, APSI, MLSI) in studied females. It may be related to the fact that the studied females were physically active, which may illustrate the relationship between physical activity and balance, including significantly lower falls risk. Effect of physical activity (multicomponent training) on elderly’s functional fitness was proven by Forte, Neiva & Marinho (2021). These findings can be also confirmed by the study by Wołoszyn, Wiśniowska-Szurlej & Sozański (2018) on relationship of physical activity and risk of falls in adults over 60 years of age. Our observation may be also related to survey’s participants muscle mass, whose relationship with balance level was observed by Knahal et al. (2021).

## Conclusions

The conducted study provides interesting information for research purposes, and can be used in therapeutic practice. Therapeutic programmes dedicated to older adults should therefore predominantly consist of strengthening exercise in the essential movement planes—the sagittal and the coronal planes. The most accurate predictor of falls in studied women was the overall stability index in tests with eyes closed. On this basis we may conclude that there is a need to include exercise performed with eyes closed in intervention programmes after having developed an adequate postural control with eyes open. This observation may also suggest that functional tests which aim at determining balance or risk of falls performed with eyes closed may point to certain disorders earlier or more sensitively.

We are conscious of some limitations of our study; they concern mainly the fact that participants were physically active, and we cannot directly associate our findings to the wide population. Balance performance is also related to the training (active life style and effect of learning). It should be taken into account when calculating the risk of falls using balance timed test.

## Supplemental Information

10.7717/peerj.12952/supp-1 Supplemental Information 1 Raw data

## Additional Information and Declarations

### Data Availability

The following information was supplied regarding data availability: The raw measurements are available in the Supplementary File.
